# A Longitudinal Study on the Effect of Exercise Habits on Locomotive Syndrome and Quality of Life during the Coronavirus Disease 2019 Pandemic

**DOI:** 10.3390/jcm13051385

**Published:** 2024-02-28

**Authors:** Sadayuki Ito, Hiroaki Nakashima, Naoki Segi, Jun Ouchida, Ryotaro Oishi, Ippei Yamauchi, Shinya Ishizuka, Yasuhiko Takegami, Taisuke Seki, Yukiharu Hasegawa, Shiro Imagama

**Affiliations:** 1 Department of Orthopedic Surgery, Nagoya University Graduate School of Medicine, Nagoya 466-8550, Japan; sadaito@med.nagoya-u.ac.jp (S.I.); segi.naoki@med.nagoya-u.ac.jp (N.S.); ouchida@med.nagoya-u.ac.jp (J.O.); ryo.oishi@med.nagoya-u.ac.jp (R.O.); yaip0411@med.nagoya-u.ac.jp (I.Y.); shinyai@med.nagoya-u.ac.jp (S.I.); takegami@med.nagoya-u.ac.jp (Y.T.); imagama@med.nagoya-u.ac.jp (S.I.); 2 Department of Orthopedic Surgery, Aichi Medical University Medical Center, Okazaki 444-2148, Japan; taiseki@aichi-med-u.ac.jp; 3 Department of Rehabilitation, Kansai University of Welfare Science, Kashiwara 582-0026, Japan; hasegawa@tamateyama.ac.jp

**Keywords:** exercise habits, quality of life, locomotive syndrome, COVID-19

## Abstract

During the COVID-19 pandemic, this study investigated the potential of exercise habits to improve quality of life (QOL) and prevent locomotive syndrome (LS) in residents of Yakumo-cho, Hokkaido, Japan. Participants from the 2018 health checkup were surveyed in February 2022, focusing on 200 respondents. These individuals were divided based on their 2018 exercise habits (at least 1 h per week): the exercise group (E group) and the non-exercise group (N group), further categorized in 2022 into the 2022E and 2022N groups. QOL was measured using the SF-36 (physical functioning, general health, physical role, physical pain, vitality, social functioning, emotional role, and mental health) and EuroQoL 5-dimension 5-level questionnaires (mobility, self-care, usual activities, pain/discomfort, and anxiety/depression), and LS was assessed with the 25-question geriatric locomotive function scale. These showed no significant change in exercise habits from 2018 to 2022. In the non-LS group, the 2022E group had higher vitality and emotional role functioning scores compared to the 2022N group. For those with LS, the 2022E group reported less physical pain. Notably, the LS incidence was significantly lower in the 2022E group. This study concludes that consistent exercise habits positively impact QOL and reduce the LS risk, underscoring the importance of regular physical activity, especially during challenging times like a pandemic. These findings highlight the broader benefits of maintaining exercise routines for public health, particularly in periods of global health crises. Based on our findings, we recommend that people continue to exercise at least one hour per week to prevent LS.

## 1. Introduction

The novel coronavirus disease 2019 (COVID-19) pandemic has drastically reshaped our living environment and necessitated adjustments to our daily activities [1]. In the years following the initial COVID-19 outbreak, individuals have continued to combat its spread by adopting social-distancing strategies, such as refraining from outdoor activities, embracing teleworking, and implementing school closures. These changes have significantly affected individuals’ lifestyles, particularly physical activity levels [2]. Recently, people are gradually resuming outdoor activities with the decline in COVID-19 cases. However, social activity appears to be evolving with COVID-19. Therefore, behavioral changes are required to improve existing exercise and lifestyle habits and shift to a new lifestyle.

Decreased physical activity directly affects health status and quality of life (QOL) [3]. Physical inactivity increases the risk of locomotive syndrome (LS), especially in the elderly [4]. LS is a condition characterized by a decline in motor function, making independent living challenging, and it has gained global attention as an assessment of motor function in locomotive diseases [5,6]. This condition decreases QOL and increases the socioeconomic burden [7].

Amid the COVID-19 pandemic, understanding how individual exercise habits change and their effects on LS and QOL within the context of restricted lifestyles aimed at infection prevention is crucial [8]. When considering societal measures for future lifestyle changes, a deeper investigation into the relationship between exercise habits and these factors is necessary.

Previous cross-sectional studies have illuminated the role of exercise in preventing LS and improving QOL [9,10]. However, these studies have primarily been conducted under normal circumstances and have not explored how drastic lifestyle changes, such as those brought about by the COVID-19 pandemic, may affect this dynamic. Therefore, a longitudinal study on the effect of exercise habits on LS and QOL before and after the onset of the COVID-19 pandemic could help bridge this research gap. Identifying the importance and potential benefits of regular exercise can inform future interventions for improving physical and social health.

Focusing on exercise habits during the COVID-19 pandemic is a unique challenge posed by social-distancing and lifestyle restrictions. Because this study focuses on the impact of the pandemic, it can provide unique insights into adaptive behaviors that can maintain or improve health status in the face of global disruption. Understanding these dynamics is critical to developing effective public health strategies and interventions in similar future scenarios.

This study seeks to investigate how changes in exercise habits due to the COVID-19 pandemic have affected individuals’ quality of life and susceptibility to locomotive syndrome. The primary hypothesis posits that maintaining or adapting exercise routines during the pandemic has mitigated the negative impacts on QOL and LS compared to pre-pandemic levels.

This research aims to contribute to the existing literature by providing a longitudinal perspective on the relationship between exercise habits and health outcomes during the COVID-19 pandemic. Its novelty lies in examining the effects of pandemic-induced lifestyle changes on physical activity and its subsequent impact on health and well-being. This study fills a critical gap by assessing the long-term implications of altered exercise habits during an unprecedented global health crisis.

## 2. Materials and Methods

### 2.1. Study Participants

**Figure 1:**
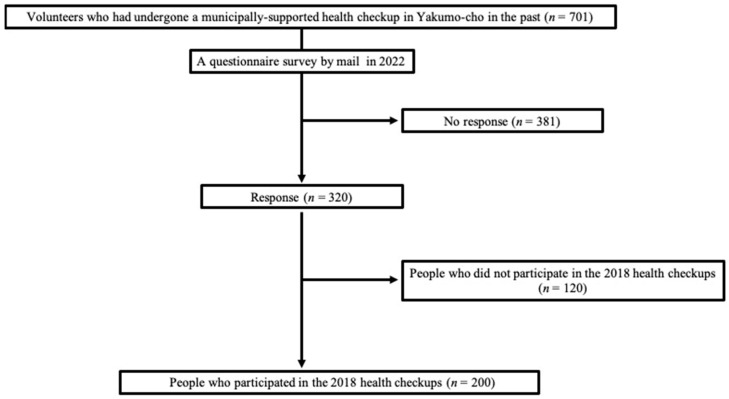
Flowchart of the patient selection.

In Yakumo-cho, Hokkaido, annual health checkups have been conducted since 1982, including voluntary orthopedic examinations, physical function tests, internal examinations, psychological tests, and health-related QOL surveys. Participants are notified by mail of the physical examinations, and those who wish to take the examinations are required to do so at a designated time each year. In order to obtain accurate test results, each item is measured by a team of specialists in each field. Yakumo-cho has a population of approximately 17,000, with 35% aged ≥65 years. Compared with residents in urban areas, those in rural areas, such as Yakumo-cho, engage more in agriculture and fishing [11]. However, owing to the COVID-19 pandemic in 2020, the health checkups for 2020 and 2021 were canceled. A questionnaire survey was conducted in 2022 via mail, involving 701 volunteers who had previously participated in the municipally supported health checkups in Yakumo-cho, to investigate the effects of the COVID-19 pandemic. Among the respondents, all the participants from the 2018 health examinations who underwent orthopedic musculoskeletal examinations and completed an LS risk-stage assessment were included. The exclusion criteria were missing data and an incomplete response to the questionnaire. The Human Research Ethics Committee and our Institutional Review Board approved the research protocol. All the participants provided written informed consent before participation. This study was conducted in accordance with the principles of the Declaration of Helsinki. Of the 701 participants to whom questionnaires were mailed, 320 completed the questionnaire, of which 200 (95 men and 105 women) met the inclusion criteria (Figure 1).

### 2.2. The 2018 Health Checkup

#### Bioelectrical Impedance Analysis (BIA)

Anthropometric data, including height, weight, body mass index (BMI), and skeletal muscle mass index (SMI) for each limb, were measured using BIA. An InBody 770 BIA device (Inbody Co., Ltd., Seoul, Republic of Korea), which can differentiate tissues based on their electrical impedance, was used [12]. The BMI was calculated using the formula: weight (kg)/height^2^ (m^2^). The BIA device automatically calculated the muscle mass of each limb. The SMI was calculated using the formula: SMI  =  appendicular skeletal muscle mass (kg)/height^2^ (m^2^).

### 2.3. Motor Function Examination

#### 2.3.1. Back Muscle Strength

Back muscle strength was defined as the maximum isometric force generated by the back extensor muscles, as measured using a digital T.K.K. 5102 dynamometer (Takei Seisakusho, Tokyo, Japan) in a standing position with lumbar flexion of 30° and knee extension. The mean forces from the two trials were recorded, and the maximum force in each trial was measured [13].

#### 2.3.2. Grip Strength

Grip strength was measured in a standing position, once for each hand, using a handgrip dynamometer (Toei Light Co., Ltd., Saitama, Japan), and the mean value was used for analysis [13]. 

#### 2.3.3. Gait Speed

Mobility was assessed using a 10 m walking time test, measuring the time required for each participant to complete a 10 m straight course at their fastest pace. The participants were tested twice, and the mean time was used for analysis [12].

### 2.4. Questionnaire Survey Conducted in 2018 and 2022

#### 2.4.1. Exercise Habits

We categorized the participants who exercised at least 1 h per week as the group with exercise habits and those who did not as the group without exercise habits.

The group with exercise habits in 2018 was designated as Group 2018E, and the group without exercise habits was designated as Group 2018N. The group with exercise habits in 2022 was designated as the 2022E group, and the group without exercise habits as the 2022N group.

#### 2.4.2. LS Stage Tests

The LS risk was assessed based on the Japanese Orthopedic Association (JOA) criteria using three tests: the two-step test, rise test, and 25-question geriatric motor function rating scale (GLFS-25) [12]. These tests defined the LS stages, which are classified as stages 1 and 2. Stage 1 indicates that motor function has begun to decline, whereas Stage 2 indicates that motor function is progressing in the direction of decline.

We used only the GLFS, which can be determined via questionnaire 25, to determine the LS stage.

The GLFS-25 is a comprehensive self-report survey that refers to the previous month. The scale comprises four questions on pain, sixteen questions on activities of daily living, three questions on social functioning, and two questions on mental status. Each item is graded from no impairment (0 points) to severe impairment (4 points).

The LS stages were defined as follows:

- LS0: A 25-question GLFS score < 7.
- LS1: A 25-question GLFS score ≥ 7.
- LS2: A 25-question GLFS score ≥ 16.

We classified the participants with LS0 as normal (NLS) and those with LS1 and 2 as participants with LS (LS). The group classified as normal in 2018 was designated as 2018NLS, and the group classified as LS was designated as 2018LS. The group classified as normal in 2022 was designated as 2022NLS, and the group classified as LS was designated as 2022LS.

#### 2.4.3. QOL

##### SF-36 QOL Questionnaire [14,15]

The SF-36 is a commonly used health-related QOL scale worldwide and has been validated in various languages. It is a multicultural scale comprising 36 questions classified into 8 domains: physical functioning (PF: 10 items), general health (5 items), physical role (RP: 4 items), physical pain (BP: 2 items), vitality (VT: 4 items), social functioning (SF: 2 items), emotional role (RE: 3 items), and mental health (MH: 5 items). Each domain is rated on a scale from 0 to 100, with higher scores indicating better health status. 

Based on the eight subcomponent scores, three summary component scores were calculated: physical component score, indicating physical health; role and social component score (RCS), indicating physical and mental health roles in professional and household activities and participation in social life; and mental component score, indicating emotional health performance, such as MH and VT [16].

##### The EuroQoL 5-Dimension 5-Level (EQ-5D-5L) [17]

The EQ-5D-5L is the latest version of the EQ-5D multi-attribute health classification system for measuring health-related QOL and utility scores. It comprises five dimensions: mobility, self-care, usual activities, pain/discomfort, and anxiety/depression. Each dimension has five severity levels: no, mild, moderate, severe, and extreme problems. The utility score is, in principle, a preference weight measured on a radix scale from 0 to 1, with “0” meaning death and “1” meaning perfect health.

### 2.5. Statistical Analysis

Continuous variables are expressed as the mean ± standard deviation. We compared the continuous variables between the E and N groups using Student’s *t*-test and the Mann–Whitney U test, depending on the items. Levene’s test for equal variances was performed with the Mann–Whitney U test if *p* < 0.05; otherwise, Student’s *t*-test was used. The continuous variables for which Student’s *t*-test was performed were listed with Cohen’s d as the effect size. The continuous variables for which Mann–Whitney tests were performed were listed with the effect sizes calculated using the automatic calculator at https://www.psychometrica.de/effect_size.html (accessed on 24 February 2024). The categorical variables were compared between the groups using the chi-square test. The categorical variables for which the chi-square test was performed were listed with Phi as the effect size. 

A logistic regression analysis was performed to separately evaluate the important factors in the 2022E group for the 2018N and 2018E groups. The dependent variable was 2022N vs. 2022E. Following univariate analysis, the variables that yielded a *p*-value < 0.05 were included in the multivariate analysis. In the analysis of 2022E for both the 2018N and 2018E groups, gender and age were included in the analysis because they are important factors for exercise. The 2022E for 2018N group included RE and RCS, which were significantly different in the univariate analysis. The 2022E for the E group included RE and RCS, which were significantly different in the univariate analysis. In 2022E in the group 2018E, we added 2018PF, 2022PF, 2022RP, 2022VT, 2022SF, 2022RE, 2022MH, 2022EQ-5D-5L, and 2022LS, which showed significant differences in the univariate analysis.

All the statistical analyses were performed using SPSS Statistics software (version 29.0; IBM Corp., Armonk, NY, USA). Statistical significance was set at *p* < 0.05.

## 3. Results

### 3.1. Exercise Habit

Among the participants in the 2018 health checkup, 44% had an exercise habit.

**Figure 2:**
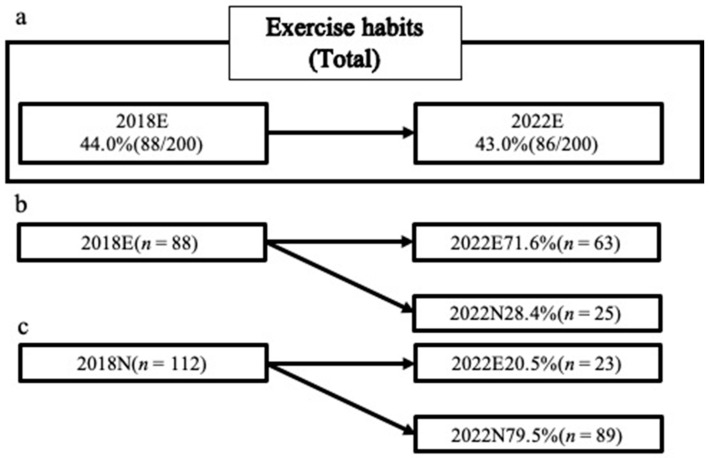
The ratio of exercise habits in 2018 and 2022. 2018N group: the group without exercise habits in 2018; 2018E group: the group with exercise habits in 2018; 2022N group: the group without exercise habits in 2022; 2022E group: the group with exercise habits in 2022. (**a**): Comparison of all participants; the percentage of participants with exercise habits in 2018 and 2022, (**b**): Comparison for participants with exercise habits in 2018; the percentage of participants with and without exercise habits in 2022, (**c**): Comparison for participants without exercise habits in 2018; the percentage of participants with and without exercise habits in 2022.

The percentage of participants with an exercise habit in 2022 was 43%. No significant differences in the percentages of participants with an exercise habit were observed between 2018 and 2022. Among the participants who had an exercise habit in 2018, 71.6% continued to exercise in 2022. Among the participants who did not have an exercise habit in 2018, 20.5% had developed an exercise habit by 2022 (Figure 2).

### 3.2. LS and Exercise Habit

Table 1 presents the participants’ characteristics in 2018. The participants had an average age of 66.5 ± 9.1 years, with 95 men and 105 women. There were 132 participants in the NLS group and 68 in the LS group. 

### 3.3. Participants without LS in 2018 (NLS)

**Figure 3:**
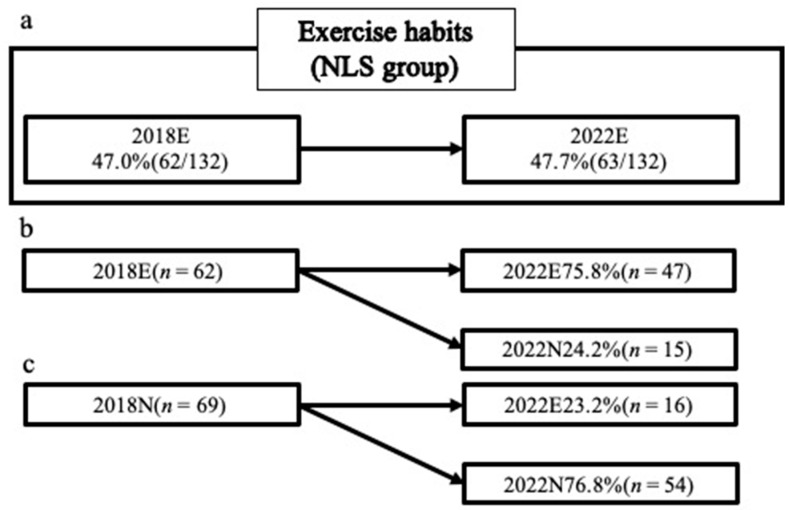
The ratio of exercise habits in the NLS group in 2018 and 2022. NLS group: the group without LS in 2018; 2018N group: the group without exercise habits in 2018; 2018E group: the group with exercise habits in 2018; 2022N group: the group without exercise habits in 2022; 2022E group: the group with exercise habits in 2022. (**a**): Comparison of the participants in the NLS group; the percentage of participants with exercise habits in the NLS group in 2018 and 2022, (**b**): Comparison for participants with exercise habits in the NLS group in 2018; the percentage of participants with and without exercise habits in 2022, (**c**): Comparison for participants without exercise habits in the NLS group in 2018; the percentage of participants with and without exercise habits in 2022.

Among the NLS group, 47% had exercise habits in 2018 and 47.7% in 2022. No significant differences in the percentages of participants with an exercise habit were observed between 2018 and 2022. Among the participants who had an exercise habit in 2018, 75.8% maintained it in 2022. Of the participants without an exercise habit in 2018, 23.2% had developed one by 2022 (Figure 3).

The VT and RE subscales of the SF-36 were higher in the participants with an exercise habit in 2022 than in those without (Table 2). 

### 3.4. Participants with LS in 2018 (LS)

**Figure 4:**
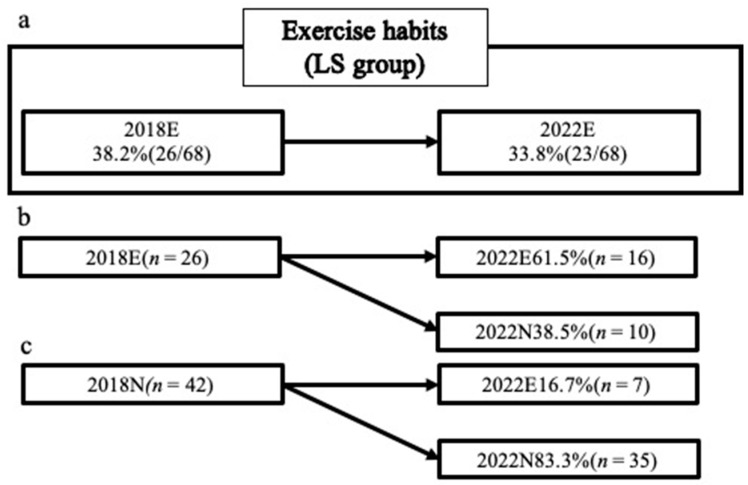
The ratio of exercise habits in the LS group in 2018 and 2022. LS group: the group with LS in 2018; 2018N group: the group without exercise habits in 2018; 2018E group: the group with exercise habits in 2018; 2022N group: the group without exercise habits in 2022; 2022E group: the group with exercise habits in 2022. (**a**): Comparison of the participants in the LS group; the percentage of participants with exercise habits in the LS group in 2018 and 2022, (**b**): Comparison for participants with exercise habits in the LS group in 2018; the percentage of participants with and without exercise habits in 2022, (**c**): Comparison for participants without exercise habits in the LS group in 2018; the percentage of participants with and without exercise habits in 2022.

Among the LS group, 38.2% had an exercise habit in 2018 and 33.8% in 2022. Among the participants with an exercise habit in 2018, 61.5% maintained it in 2022. Among the participants without an exercise habit in 2018, 16.7% had developed one by 2022 (Figure 4). The BP subscale of the SF-36 was higher in the participants with an exercise habit in 2022 than in those without (Table 3). 

### 3.5. Comparison between Groups with and without Exercise Habits

Table S1 shows the characteristics of the participants in 2018. There were 112 participants in the N group and 88 in the E group. The participants in Group E were significantly older than those in Group N (N: 65.2 ± 9.3, E: 68.1 ± 8.5, *p* = 0.024). No significant differences were observed between the E and N groups for the other items, such as QOL and LS.

In 2022, we compared the participants with and without exercise habits in Group N (participants without exercise habits in 2018 before the COVID-19 pandemic) and Group E (participants with exercise habits).

The 2022E group consisted of those with exercise habits, while the 2022N group did not have exercise habits.

### 3.6. Participants in the 2018N Group

Group 2022N had 89 participants (37 men and 52 women) with a mean age of 65.0 ± 9.0 years, and Group 2022E had 23 participants (11 men and 12 women) with a mean age of 66.0 ± 10.6 years. No significant differences in sex or age were observed between the groups.

No significant differences were observed in the muscle mass, muscle strength, SF-36, EQ-5D-5L, or LS incidence in 2018 (Table S2).

The RE subscale of the SF-36 in 2022 was significantly higher in the 2022E group than in the 2022N group. Additionally, the RCS was significantly higher in the 2022E group than in the 2022N group. No significant differences in the EQ-5D-5L and LS were observed between the 2022N and 2022E groups (Table S3).

Logistic regression analysis did not identify any significant factors in the 2022E group (Table S4).

### 3.7. Participants in the 2018E Group

Group 2022N had 25 participants (10 men and 15 women) with a mean age of 66.6 ± 9.9 years, and Group 2022E had 63 participants (37 men and 26 women) with a mean age of 68.7 ± 7.8 years. No significant differences in sex or age were observed between the groups.

Only the PF subscale of the SF-36 was significantly higher in the 2022E group than in the 2022N group in 2018. In addition to the summary score in 2018, no significant differences were observed in the other SF-36 subscales, including the muscle mass, muscle strength, SF-36, EQ-5D-5L, and LS incidence (Table S5).

The PF, RP, VT, SF, RE, and MH subscales of the SF-36 were significantly higher in the 2022E group than in the 2022N group in 2022. Additionally, the EQ-5D-5L was significantly higher in the 2022E group than in the 2022N group. Furthermore, the LS incidence was significantly lower in the 2022E group than in the 2022N group (N: 92%, E: 44.4%, *p* < 0.001) (Table S6).

We examined 2018PF, 2022PF, 2022RP, 2022VT, 2022SF, 2022RE, 2022MH, 2022 EQ-5D-5L, and 2022LS as covariates for important factors for the 2022E group in the logistic regression analysis. The results revealed that only 2022LS was an important factor in the 2022E group (Exp(B) 0.123, 95% confidence interval: 0.021–0.738, *p* = 0.022) (Table S7).

## 4. Discussion

This study aimed to investigate the impact of exercise habits on locomotive syndrome (LS) and quality of life (QOL) during the COVID-19 pandemic. Our findings indicate that maintaining exercise habits during the pandemic is associated with better QOL and may help mitigate the risk of LS, aligning with the study’s objectives. Previous studies have indicated that consistent physical activity positively influences LS development and overall QOL [9,10]. Our study deepens and expands on these findings, considering the unique circumstances of the pandemic. When participants diagnosed with LS before the COVID-19 pandemic were compared based on their exercise habits during the pandemic, no significant difference was observed in the LS prevalence. However, some aspects of QOL were better in the group with exercise habits. Similarly, no significant difference was observed in the LS prevalence among participants without LS during the COVID-19 pandemic. However, some aspects of QOL were better in the group with exercise habits. Participants were classified based on their exercise habits before the COVID-19 pandemic and compared longitudinally based on their exercise habits during the COVID-19 pandemic. The results showed that those who already had an exercise habit either continued or discontinued it, with the latter group showing a significantly increased rate of LS and decreased QOL. However, in the group that did not exercise, the results showed that compared with the group that did not commence exercise during the pandemic, the group that did improved their social-related QOL.

This study’s strengths lie in its longitudinal design and focus on the impact of exercise habits on locomotive syndrome (LS) and quality of life (QOL) during the unprecedented global health crisis of the COVID-19 pandemic. One of the key aspects contributing to the robustness of our research is the utilization of a well-defined rural population, which, despite its limitations in generalizability, provided a unique opportunity to observe changes in exercise habits and their effects in a relatively homogeneous and stable environment. This specificity allows for a clearer understanding of the relationship between exercise and health outcomes in the context of pandemic-related lifestyle changes. Another significant strength is the study’s timing, capturing data before and during the COVID-19 pandemic. This longitudinal approach offers valuable insights into how sudden global events can influence health behaviors and outcomes, contributing to the literature on public health and crisis management. Furthermore, the study’s focus on both LS and QOL addresses a gap in the existing research, particularly regarding the social aspects of QOL, which are often overlooked in physical health studies. The inclusion of various health-related measures, such as the SF-36 QOL questionnaire and the EuroQoL 5-dimension 5-level (EQ-5D-5L), enhances the study’s robustness by providing a comprehensive view of participants’ health status. This multi-dimensional approach allows for a more nuanced understanding of how exercise habits impact different facets of health and well-being.

The COVID-19 pandemic considerably influenced human behavior, including exercise habits. Our findings suggest that the proportion of individuals with exercise routines remained constant before and during the pandemic. This consistency in physical activity contributed to better QOL during the pandemic, regardless of their LS status before the pandemic. The existing literature has established that exercise contributes significantly to physical and mental well-being [18]. During challenging times, such as the COVID-19 pandemic, physical activity has served as a protective factor against psychological distress [19]. The importance of routines is also paramount, especially during disruptions such as pandemics [20]. Maintaining an exercise routine can offer stability and normalcy that effectively enhance QOL. This finding is compelling in individuals with LS. Previous studies have posited the positive effects of exercise in delaying the progression and mitigating LS symptoms [4]. Our observation that these individuals reported good QOL when maintaining an exercise regimen further underscores the universal benefits of physical activity.

The results of this study underscore the importance of individuals with an exercise habit continuing their routines during the COVID-19 pandemic to maintain their QOL and prevent LS. It has been reported that adequate preparation is beneficial to performance in top-level athletes [21]. This reaffirms the importance of sustained exercise for preventing LS [22] and maintaining QOL [3].

The group that discontinued exercise had significantly lower QOL and higher LS rates, confirming previous findings that exercise is important for maintaining and improving physical function [3]. Moreover, our results confirmed that appropriate exercise habits may reduce LS occurrence in the elderly [23]. The results also confirmed that the health benefits were sustained when exercise was continued. This suggests that continuous exercise maintains physical function, improves the QOL, and decreases the risk of certain diseases [24]. Thus, the importance of continuing exercise habits during the COVID-19 pandemic is demonstrated, suggesting the need for social support and environmental arrangements. 

In contrast, for individuals without exercise habits, those who started exercising during the pandemic showed improved social-related QOL than those who did not. This finding indicates that exercise may improve QOL in the social dimension by facilitating social communication and interaction, reducing isolation, and increasing self-efficacy [25]. The primary novelty of our study stems from its findings related to the social aspects of QOL. Previous studies have primarily focused on the physical benefits of exercise [3]. Our research expands beyond this scope by suggesting that physical activity can also have significant social benefits. However, initiating exercise did not yield significant differences for other aspects of QOL and LS. This is because the effects on physical function and QOL may not immediately manifest when adopting a new exercise routine [26]. Furthermore, given that the formation and continuation of exercise habits are necessary for preventing LS, these effects would not appear in a short period [22]. Thus, these results suggest that while exercise habits immediately benefit social health, the formation and continuation of exercise habits over time is necessary to realize other health benefits.

Furthermore, our study uniquely evaluates these effects within the context of the COVID-19 pandemic, a global event that dramatically altered daily routines, including exercise habits. Our study provides important insights into how changes in exercise habits during significant life events or crises can affect physical and social health.

However, it is crucial to consider alternative explanations and acknowledge the uncertainties in our interpretation. One alternative explanation for the observed benefits of exercise for QOL and LS could be the inherent health consciousness of participants who regularly engage in physical activity. These individuals might have other healthy behaviors that contribute to their overall well-being, suggesting that exercise is part of a broader lifestyle pattern. Additionally, the social support systems available to individuals who maintain exercise habits, even during the pandemic, could play a significant role in their perceived QOL.

This study, while providing valuable insights into the effects of exercise habits on quality of life and locomotive syndrome during the COVID-19 pandemic, has several limitations that warrant consideration. Firstly, the study’s participants were primarily middle-aged and older adults residing in relatively rural areas, many of whom were engaged in occupations such as farming and fishing. This demographic and geographical focus may limit the generalizability of our findings to broader populations, especially those living in urban settings with different lifestyle patterns and environmental factors. The unique physical demands and social structures of rural living could influence exercise habits and health outcomes differently compared to urban counterparts, potentially affecting the applicability of our results to a wider audience. Secondly, the reliance on self-reported exercise habits introduces the possibility of reporting biases, which could affect the accuracy of the data collected. Participants may overestimate their physical activity levels or fail to accurately recall their exercise routines, leading to discrepancies between reported habits and actual behavior. This limitation underscores the need for objective measures of physical activity in future research to provide more reliable and precise assessments of exercise habits. Thirdly, the evolving nature of the COVID-19 pandemic presents a challenge to the study’s time frame and relevance. The pandemic’s impact on lifestyle, exercise habits, and health outcomes is dynamic, with varying degrees of restrictions and public health measures over time. Consequently, the findings of this study, conducted within a specific period, may not fully capture the long-term effects of the pandemic or reflect changes in behavior as the situation evolves. Finally, the potential influence of health consciousness among participants, as indicated by their regular attendance at annual health checkups, may also limit the study’s findings. This characteristic suggests a higher level of health awareness and motivation to engage in health-promoting behaviors than the general population, which could skew the results. Participants who are proactive about their health are likely to maintain better exercise habits and have more favorable health outcomes, potentially leading to an overestimation of the positive effects of exercise on quality of life and locomotive syndrome. Acknowledging these limitations is crucial to interpreting the study’s findings and guiding future research. Multicenter studies involving a more diverse participant pool, the use of objective measures of physical activity, and longitudinal designs that account for the pandemic’s evolving nature are recommended to validate and expand upon our findings.

## 5. Conclusions

Our study contributes to the understanding of the importance of regular exercise in preventing LS and improving QOL, particularly during the COVID-19 pandemic. The findings highlight the potential benefits of maintaining exercise habits for enhancing social aspects of QOL and mitigating the risk of LS. The longitudinal design and focused examination of a rural population have unveiled nuanced insights into the resilience of exercise habits and their positive effects on physical and social well-being. These findings underscore the importance of promoting and maintaining physical activity as a key public health strategy, not only for its physical benefits but also for its significant contributions to social health and quality of life. This study’s unique contributions to understanding the impact of global crises on health behaviors and outcomes highlight the need for flexible and adaptive public health strategies that can support physical activity in various contexts. By emphasizing the social benefits of exercise, our research expands the conversation beyond traditional physical health metrics, offering a more holistic view of well-being. However, given the uncertainties arising from the study’s limitations and the possibility of alternative explanations for our findings, it is prudent to refrain from making definitive conclusions. Future research should aim to address these limitations by including a more diverse participant pool, employing objective measures of physical activity, and considering the long-term impacts of the pandemic on exercise habits and health outcomes. Such studies are essential for providing a more comprehensive understanding of the role of exercise in promoting health and well-being during and beyond the pandemic.

## Figures and Tables

**Table 1:** The comparison of each parameter in 2018 between the NLS and LS groups.

2018	Total (*n* = 200)	NLS (*n* = 132)	LS (*n* = 68)	*p*	Effect Size
Men/women	95/105	66/66	29/39	0.371	−0.070
Age (y)	66.5 ± 9.1	65.8 ± 9.1	68 ± 9.0	0.100	−0.246
Height (cm)	158.2 ± 8.2	158.3 ± 8.1	157.9 ± 8.6	0.744	0.050
Weight (kg)	59 ± 10.7	58.2 ± 10.8	60.5 ± 10.5	0.163	−0.210
BMI (kg/m^2^)	23.5 ± 3.2	23.1 ± 3.1	24.2 ± 3.3	0.023	−0.343
BFP (%)	27.8 ± 6.5	27.3 ± 6.1	28.8 ± 7.2	0.128	−0.232
SMI (kg/m^2^)	7.31 ± 1.13	7.23 ± 1.16	7.46 ± 1.07	0.334	−0.210
Right grip strength	30.2 ± 9.7	30.8 ± 10.0	29 ± 9.0	0.220	0.185
Left grip strength	29.1 ± 9.7	29.9 ± 10.2	27.6 ± 8.6	0.106	0.244
Back muscle strength (kg)	80.3 ± 34.2	79.9 ± 32.8	81 ± 37.3	0.872	−0.031
Gait speed (m/s)	2.2 ± 0.3	2.2 ± 0.3	2.1 ± 0.3	0.024	0.441
PF	88.5 ± 17.0	93.4 ± 14.0	79 ± 18.3	<0.001	−0.493
RP	87.3 ± 21.8	93.8 ± 14.4	74.7 ± 27.6	<0.001	−0.436
BP	71.2 ± 23.5	80.3 ± 19.1	53.5 ± 20.9	<0.001	1.356
GH	66.7 ± 19.7	72.5 ± 17.2	55.4 ± 19.4	<0.001	0.951
VT	60.9 ± 19.5	66.3 ± 16.9	50.3 ± 20.1	<0.001	0.883
SF	86.1 ± 20.7	90 ± 17.6	78.7 ± 24.2	<0.001	−0.257
RE	88 ± 22.6	93.9 ± 14.5	76.5 ± 30.1	<0.001	−0.335
MH	75.5 ± 18.2	79.4 ± 16.7	68.1 ± 18.8	<0.001	0.647
PCS	49.2 ± 9.3	52.8 ± 6.6	42.1 ± 9.8	<0.001	1.381
MCS	51.5 ± 9.6	53.6 ± 8.8	47.6 ± 9.8	<0.001	0.654
RCS	49.9 ± 11.3	51.2 ± 7.6	47.6 ± 16.0	0.036	−0.019
EQ-5D-5L	0.89 ± 0.13	0.93 ± 0.07	0.8 ± 0.16	<0.001	−0.537
GLFS-25	6.9 ± 9.3	2.5 ± 2.0	15.4 ± 11.8	<0.001	0.822

LS, locomotive syndrome; NLS group, the group without LS in 2018; LS group, the group with LS in 2018; BMI, body mass index; BFP, body fat percentage; SMI, skeletal muscle mass index; PF, physical functioning; GH, general health; RP, physical role; BP, physical pain; VT, vitality; SF, social functioning; RE, emotional role; MH, mental health; PCS, physical component summary; MCS, mental component summary; RCS, role/social component summary; EQ-5D-5L, EuroQoL 5-dimension 5-level; GLFS-25, 25-question geriatric motor function rating scale.

**Table 2:** The comparison of each parameter between the 2022N and 2022E groups in the NLS group.

	NLS (*n* = 132)	2022N (*n* = 69)	2022E (*n* = 63)	*p*	Effect Size
2018					
Men/women	66/66	30/39	36/27	0.163	0.137
Age (y)	65.8 ± 9.1	64.6 ± 9.4	67 ± 8.6	0.133	−0.265
Height (cm)	158.3 ± 8.1	157.1 ± 8.1	159.7 ± 8.0	0.069	−0.32
Weight (kg)	58.2 ± 10.8	56.5 ± 10.1	60.1 ± 11.3	0.052	−0.342
BMI (kg/m^2^)	23.1 ± 3.1	22.8 ± 3.1	23.4 ± 3.1	0.241	−0.205
BFP (%)	27.3 ± 6.1	27.7 ± 6.6	26.9 ± 5.6	0.437	0.136
SMI (kg/m^2^)	7.23 ± 1.16	7.31 ± 1.16	7.14 ± 1.16	0.554	0.149
Right Grip strength	30.8 ± 10.0	29.7 ± 10.5	31.9 ± 9.5	0.215	−0.218
Left Grip strength	29.9 ± 10.2	28.9 ± 10.4	31.1 ± 9.9	0.230	−0.21
Back muscle strength (kg)	79.9 ± 32.8	76.3 ± 30.6	83.6 ± 34.9	0.328	−0.22
Gait speed (m/s)	2.2 ± 0.3	2.2 ± 0.4	2.2 ± 0.3	0.552	−0.133
2022					
PF	90.1 ± 13.4	88.5 ± 16.5	92 ± 8.4	0.134	0.087
RP	90 ± 16.9	88.1 ± 19.5	92 ± 13.4	0.189	−0.231
BP	75.2 ± 19.8	73.1 ± 19.7	77.5 ± 19.8	0.205	−0.223
GH	71.6 ± 18.8	70.3 ± 20.0	73.1 ± 17.3	0.410	−0.145
VT	67.4 ± 18.4	64.2 ± 20.2	70.9 ± 15.6	0.039	−0.366
SF	84.5 ± 24.8	80.5 ± 27.2	88.9 ± 21.3	0.053	−0.341
RE	91.6 ± 16.0	87.9 ± 19.4	95.7 ± 9.7	0.005	−0.036
MH	79.6 ± 16.1	77.1 ± 16.7	82.4 ± 15.1	0.062	−0.329
PCS	51 ± 8.7	50.9 ± 9.6	51.1 ± 7.6	0.891	−0.024
MCS	54.1 ± 9.1	53 ± 9.5	55.2 ± 8.6	0.166	−0.245
RCS	48.9 ± 10.9	47.1 ± 12.6	50.9 ± 8.2	0.051	0.005
EQ-5D-5L	0.95 ± 0.07	0.94 ± 0.07	0.95 ± 0.06	0.412	−0.144
GLFS-25	6.4 ± 4.7	6.9 ± 4.7	5.9 ± 4.6	0.237	0.207
2022LS (NLS/LS)	72/60	34/35	38/25	0.224	−0.111

LS, locomotive syndrome; NLS group, the group without LS in 2018; 2022N group, the group without exercise habits in 2022; 2022E group, the group with exercise habits in 2022; BMI, body mass index; BFP, body fat percentage; SMI, skeletal muscle mass index; PF, physical functioning; GH, general health; RP, physical role; BP, physical pain; VT, vitality; SF, social functioning; RE, emotional role; MH, mental health; PCS, physical component summary; MCS, mental component summary; RCS, role/social component summary; EQ-5D-5L, EuroQoL 5-dimension 5-level; GLFS-25, 25-question geriatric motor function rating scale; 2022LS(NLS/LS), normal group (NLS group) in 2022/LS group (LS group) in 2022.

**Table 3:** The comparison of each parameter between the 2022N and 2022E groups in the LS group.

	LS (*n* = 68)	2022N (*n* = 45)	2022E (*n* = 23)	*p*	Effect Size
2018					
Men/women	29/39	17/28	12/11	0.305	0.138
Age (y)	68 ± 9.0	66.6 ± 9.0	70.7 ± 8.4	0.070	−0.469
Height (cm)	157.9 ± 8.6	157.4 ± 8.8	158.9 ± 8.2	0.511	−0.17
Weight (kg)	60.5 ± 10.5	60.9 ± 11.2	59.7 ± 9.1	0.657	0.115
BMI (kg/m^2^)	24.2 ± 3.3	24.5 ± 3.5	23.6 ± 2.8	0.296	0.271
BFP (%)	28.8 ± 7.2	30.3 ± 7.1	26.1 ± 6.7	0.026	0.593
SMI (kg/m^2^)	7.46 ± 1.07	7.52 ± 1.19	7.34 ± 0.78	0.665	0.167
Right Grip strength	29 ± 9.0	28.2 ± 9.0	30.4 ± 9.2	0.348	−0.243
Left Grip strength	27.6 ± 8.6	26.6 ± 8.5	29.5 ± 8.6	0.196	−0.336
Back muscle strength (kg)	81 ± 37.3	79.8 ± 40.4	83.3 ± 31.6	0.785	−0.09
Gait speed (m/s)	2.1 ± 0.3	2.1 ± 0.3	1.9 ± 0.2	0.057	0.65
2022					
PF	71.8 ± 20.8	69 ± 19.7	77.2 ± 22.1	0.124	−0.399
RP	74.4 ± 25.3	71.7 ± 24.0	79.6 ± 27.3	0.222	−0.316
BP	53.3 ± 17.5	49.7 ± 15.7	60.4 ± 19.0	0.016	−0.634
GH	55.7 ± 22.3	54.2 ± 23.4	58.5 ± 20.2	0.450	−0.195
VT	53.6 ± 20.4	50.3 ± 21.2	60.1 ± 17.3	0.060	−0.49
SF	73 ± 26.8	71.7 ± 28.5	75.5 ± 23.4	0.576	−0.144
RE	76.3 ± 26.6	74.1 ± 26.8	80.8 ± 26.2	0.327	−0.253
MH	68.2 ± 20.1	66 ± 20.3	72.5 ± 19.3	0.206	−0.327
PCS	38.8 ± 13.5	37 ± 13.3	42.1 ± 13.5	0.145	−0.38
MCS	49.6 ± 10.8	48.7 ± 10.2	51.2 ± 11.9	0.370	−0.232
RCS	45.7 ± 15.5	45.1 ± 16.0	46.7 ± 14.9	0.704	−0.098
EQ-5D-5L	0.82 ± 0.17	0.8 ± 0.18	0.86 ± 0.13	0.168	−0.358
GLFS-25	19 ± 15.0	20.8 ± 17.1	15.6 ± 9.2	0.180	0.347
2022LS (N/L)	8/68	4/41	4/19	0.429	−0.125

LS, locomotive syndrome; LS group, the group with LS in 2018; 2022N group, the group without exercise habits in 2022; 2022E group, the group with exercise habits in 2022; BMI, body mass index; BFP, body fat percentage; SMI, skeletal muscle mass index; PF, physical functioning; GH, general health; RP, physical role; BP, physical pain; VT, vitality; SF, social functioning; RE, emotional role; MH, mental health; PCS, physical component summary; MCS, mental component summary; RCS, role/social component summary; EQ-5D-5L, EuroQoL 5-dimension 5-level; GLFS-25, 25-question geriatric motor function rating scale; 2022LS(N/L), normal group (N group) in 2022/LS group (L group) in 2022.

## Data Availability

The health-checkup data used to support the findings of this study are available from the corresponding author upon request.

## Supplementary Materials

The following supporting information can be downloaded at https://www.mdpi.com/article/10.3390/jcm13051385/s1, Table S1: The comparison of each parameter in 2018 between the N and E groups; Table S2: Comparison of parameters between the 2022N and 2022E groups in the N group; Table S3: Comparison of each parameter in 2022 between the 2022N and 2022E groups in the L group; Table S4: Logistic regression analysis of important factors in the 2022E group in the N group; Table S5: Comparison of each parameter in 2018 between the 2022N and 2022E groups in the E group; Table S6: Comparison of each parameter in 2022 between the 2022N and 2022E groups in the E group; Table S7: Logistic regression analysis of the important factors in the 2022E group in the E group.
