# Supplementary material for: A Longitudinal Study on the Effect of Exercise Habits on Locomotive Syndrome and Quality of Life during the Coronavirus Disease 2019 Pandemic
Source: J Clin Med. 2024 Feb 28;13(5):1385. doi: 10.3390/jcm13051385 (PMC10932004; doi:10.3390/jcm13051385)
Supplement: Supplementary file 1 [file jcm-13-01385-s001.zip › jcm-2859132-supplementary.pdf]

Supplementary materials

Table S1. The comparison of each parameter in 2018 between the N and E groups

2018	Total (n=200)	N (n=112)	E (n=88)	p	Cohen's d
Men/women	95/105	48/64	47/41	0.138	
Age (yrs)	66.5±9.1	65.2±9.3	68.1±8.5	0.024	-0.325
Height (cm)	158.2±8.2	157.7±8.4	158.8±8.0	0.355	-0.132
Weight (kg)	59±10.7	58.8±10.6	59.2±10.9	0.769	-0.042
BMI (kg/m^2)	23.5±3.2	23.5±3.3	23.4±3.1	0.702	0.055
BFP (%)	27.8±6.5	28.4±7.0	27.1±5.9	0.154	0.206
SMI (kg/m^2)	7.31±1.13	7.35±1.14	7.24±1.12	0.650	0.095
Right Grip strength	30.2±9.7	29.2±9.8	31.4±9.5	0.109	-0.23
Left Grip strength	29.1±9.7	28.3±9.7	30.3±9.7	0.148	-0.208
Back muscle strength (kg)	80.3±34.2	82.7±33.3	77.1±35.5	0.38	0.162
Gait speed (m/s)	2.2±0.30	2.2±0.36	2.12±0.27	0.211	0.231
PF	88.5±17.0	86.7±15.6	90.7±18.5	0.105	-0.232
RP	87.3±21.8	87±22.5	87.7±21.0	0.818	-0.033
BP	71.2±23.5	70.9±24.2	71.5±22.6	0.837	-0.029
GH	66.7±19.7	66.5±19.2	66.9±20.4	0.875	-0.022
VT	60.9±19.5	58.5±19.0	63.8±19.9	0.057	-0.272

SF	86.1±20.7	86.8±22.1	85.2±18.9	0.588	0.077
RE	88±22.6	88.2±23.7	87.7±21.4	0.875	0.022
MH	75.5±18.2	74.4±18.8	76.9±17.4	0.342	-0.136
PCS	49.2±9.3	48.7±9.3	49.9±9.3	0.364	-0.131
MCS	51.5±9.6	50.9±9.4	52.3±9.8	0.306	-0.148
RCS	49.9±11.3	50.7±11.2	49±11.3	0.293	0.152
EQ-5D-5L	0.89±0.13	0.89±0.12	0.89±0.13	0.778	0.04
GLFS-25	6.9±9.3	7.6±9.7	6±8.8	0.241	0.168
LS (NLS/LS)	132/68	70/42	62/26	0.238	

N group, the group without exercise habits in 2018; E group, the group with exercise habits in 2018; BMI, body mass index; BFP, body fat percentage; SMI, skeletal muscle mass index, PF, physical functioning; GH, general health; RP, physical role; BP, physical pain; VT, vitality; SF, social functioning; RE, emotional role; MH, mental health; PCS, physical component summary; MCS, mental component summary; RCS, role/social component summary; EQ-5D-5L, EuroQoL 5-dimension 5-level; GLFS-25, 25-question geriatric motor function rating scale; LS, locomotive syndrome; LS(NLS/LS), normal group (NLS group)/LS group (LS group)

Table S2. Comparison of parameters between the 2022N and 2022E groups in N group

2018	N (n=112)	2022N (n=89)	2022E (n=23)	p	Cohen's d
Men/women	48/64	37/52	11/12	0.589	
Age (yrs)	65.2±9.3	65±9.0	66±10.6	0.674	-0.099
Height (cm)	157.7±8.4	157.5±8.4	158.4±8.6	0.684	-0.095
Weight (kg)	58.8±10.6	58.6±10.6	59.3±11.0	0.792	-0.062
BMI (kg/m^2)	23.5±3.3	23.6±3.3	23.5±3.1	0.968	0.009
BFP (%)	28.4±7.0	28.7±7.0	27.1±6.9	0.320	0.239
SMI (kg/m^2)	7.35±1.14	7.39±1.14	7.2±1.15	0.594	0.169
Right Grip strength	29.2±9.8	29.2±9.8	29.2±10.0	0.972	-0.008
Left Grip strength	28.3±9.7	28±9.6	29.3±10.1	0.566	-0.135
Back muscle strength (kg)	82.7±33.3	80.7±34.4	88.8±30.2	0.390	-0.242
Gait speed (m/s)	2.2±0.36	2.21±0.35	2.16±0.42	0.570	0.159
PF	86.7±15.6	86.6±14.2	87.3±20.6	0.357	-0.049
RP	87±22.5	86±22.4	90.8±23.1	0.163	-0.21
BP	70.9±24.2	68.9±24.2	78.4±23.4	0.050	-0.397
GH	66.5±19.2	66.7±19.4	65.7±18.9	0.834	0.054
VT	58.5±19	57.9±19.3	61.1±17.8	0.293	-0.172

SF	86.8±22.1	86.1±21.7	89.7±23.7	0.190	-0.162
RE	88.2±23.7	87.5±23.9	91.3±23.1	0.306	-0.162
MH	74.4±18.8	74.1±18.1	75.7±21.6	0.464	-0.081
PCS	48.7±9.3	48.1±9.6	51.1±7.6	0.279	-0.329
MCS	50.9±9.4	50.6±10	52.1±6.5	0.535	
RCS	50.7±11.2	50±11.6	53.5±9.3	0.062	-0.316
EQ-5D-5L	0.89±0.12	0.89±0.13	0.92±0.07	0.395	-0.258
GLFS-25	7.6±9.7	8.1±10.5	5.4±5.2	0.344	0.288
LS (NLS/LS)	70/42	54/35	16/7	0.432	

N group, the group without exercise habits in 2018; 2022N group, the group without exercise habits in 2022; 2022E group, the group with exercise habits in 2022; BMI, body mass index; BFP, body fat percentage; SMI, skeletal muscle mass index, PF, physical functioning; GH, general health; RP, physical role; BP, physical pain; VT, vitality; SF, social functioning; RE, emotional role; MH, mental health; PCS, physical component summary; MCS, mental component summary; RCS, role/social component summary; EQ-5D-5L, EuroQoL 5-dimension 5-level; GLFS-25, 25-question geriatric motor function rating scale; LS, locomotive syndrome; LS(NLS/LS), normal group (NLS group)/LS group (LS group)

Table S3. Comparison of each parameter in 2022 between the 2022N and 2022E groups in the N group

2022	N	2022N	20222E	p	Cohen's d
PF	82.9±18.4	82.1±19.6	86±12.6	0.792	-0.215
RP	84.6±20.8	83.2±21.7	89.9±16	0.184	-0.325
BP	65±21.1	63.6±20.9	70.4±21.6	0.234	-0.327
GH	64.9±21.0	64.5±22.1	66.7±16.3	0.807	
VT	60.8±21.3	59.1±22.7	67.4±13.2	0.133	
SF	82.4±25.3	80.5±26	89.7±21.2	0.065	-0.367
RE	86.2±21.3	83.5±22.8	96.4±8.6	0.009	-0.621
MH	74.7±18.8	72.9±19.7	81.8±12.7	0.060	-0.479
PCS	45.6±11.5	45.6±11.7	45.6±11.3	0.873	-0.002
MCS	51.7±9.9	51.2±10.4	53.8±7.3	0.341	
RCS	48.9±12.3	47.6±13.0	53.6±7.7	0.026	
EQ-5D-5L	0.9±0.12	0.9±0.13	0.92±0.10	0.327	-0.219
GLFS-25	11.2±11.0	11.3±11.9	10.5±6.2	0.552	0.077

2022LS (NLS/LS)	43/69	36/53	7/16	0.379

N group, the group without exercise habits in 2018; 2022N group, the group without exercise habits in 2022; 2022E group, the group with exercise habits in 2022; PF, physical functioning; GH, general health; RP, physical role; BP, physical pain; VT, vitality; SF, social functioning; RE, emotional role; MH, mental health; PCS, physical component summary; MCS, mental component summary; RCS, role/social component summary; EQ-5D-5L, EuroQoL 5-dimension 5-level; GLFS-25, 25-question geriatric motor function rating scale; LS, locomotive syndrome; LS(NLS/LS), normal group (NLS group)/LS group (LS group)

Table S4. Logistic regression analysis of important factors in the 2022E group in the N group

N group	B	SE	Wald	df	p	Exp(B)	95% CI
Sex	-0.158	0.51	0.096	1	0.756	0.854	0.314–2.318
Age	0.025	0.028	0.794	1	0.373	1.026	0.970–1.084
RE	0.052	0.028	3.577	1	0.059	1.054	0.998–1.112
RCS	0.004	0.038	0.012	1	0.913	1.004	0.932–1.081

N group, the group without exercise habits in 2018; 2022E group, the group with exercise habits in 2022; RE, emotional role; RCS, role/social component summary.

Table S5. Comparison of each parameter in 2018 between the 2022N and 2022E groups in the E group

2022	E	2022N	20222E	p	Cohen's d
PF	85.1±18.5	76.2±22.1	88.7±15.7	<0.001	
RP	84.7±22.4	76±25.9	88.2±19.9	0.024	
BP	71.2±22.0	64.9±23.9	73.8±20.8	0.105	-0.274
GH	67.9±21.8	62.5±25.2	70±20.1	0.213	-0.12
VT	65±18.3	57.3±17.2	68.1±18.0	0.009	-0.145
SF	78.3±26.9	64.1±31.6	83.7±23.0	0.003	-0.352
RE	86.7±21.8	78.7±26.1	89.9±19.1	0.038	
MH	76.9±17.8	72±16.3	78.9±18.1	0.033	-0.096
PCS	48.4±12.5	44.8±17.6	49.8±9.8	0.470	
MCS	53.6±9.9	51.9±8.1	54.3±10.5	0.151	0.089
RCS	46.4±13.0	41.6±16.5	48.3±11.1	0.050	-0.312
EQ-5D-5L	0.9±0.13	0.84±0.19	0.93±0.09	0.048	
GLFS-25	10.2±11.6	16.2±16.7	7.8±7.8	<0.001	
2022LS (NS/LS)	37/51	2/23	35/28	<0.001	

E group, the group without exercise habits in 2018; 2022N group, the group without exercise habits in 2022; 2022E group, the group with exercise habits in 2022; BMI,

body mass index; BFP, body fat percentage; SMI, skeletal muscle mass index, PF, physical functioning; GH, general health; RP, physical role; BP, physical pain; VT, vitality; SF, social functioning; RE, emotional role; MH, mental health; PCS, physical component summary; MCS, mental component summary; RCS, role/social component summary; EQ-5D-5L, EuroQoL 5-dimension 5-level; GLFS-25, 25-question geriatric motor function rating scale; LS, locomotive syndrome; LS(NLS/LS), normal group (NLS group)/LS group (LS group)

Table S6. Comparison of each parameter in 2022 between the 2022N and 2022E groups in the E group

2022	2018E	2022N	2022E	p	Cohen's d
PF	85.1±18.5	76.2±22.1	88.7±15.7	<0.001	
RP	84.7±22.4	76±25.9	88.2±19.9	0.024	
BP	71.2±22.0	64.9±23.9	73.8±20.8	0.105	-0.409
GH	67.9±21.8	62.5±25.2	70±20.1	0.213	-0.346
VT	65±18.3	57.3±17.2	68.1±18.0	0.009	-0.614
SF	78.3±26.9	64.1±31.6	83.7±23.0	0.003	-0.769
RE	86.7±21.8	78.7±26.1	89.9±19.1	0.038	
MH	76.9±17.8	72±16.3	78.9±18.1	0.033	-0.394
PCS	48.4±12.5	44.8±17.6	49.8±9.8	0.470	
MCS	53.6±9.9	51.9±8.1	54.3±10.5	0.151	-0.238
RCS	46.4±13.0	41.6±16.5	48.3±11.1	0.050	
EQ-5D-5L	0.9±0.13	0.84±0.19	0.93±0.09	0.048	
GLFS-25	10.2±11.6	16.2±16.7	7.8±7.8	<0.001	
2022LS(NLS/LS)	37/51	2/23	35/28	<0.001	

E group, the group without exercise habits in 2018; 2022N group, the group without exercise habits in 2022; 2022E group, the group with exercise habits in 2022; PF, physical functioning; GH, general health; RP, physical role; BP, physical pain; VT,

vitality; SF, social functioning; RE, emotional role; MH, mental health; PCS, physical component summary; MCS, mental component summary; RCS, role/social component summary; EQ-5D-5L, EuroQoL 5-dimension 5-level; GLFS-25, 25-question geriatric motor function rating scale; LS, locomotive syndrome; LS(NLS/LS), normal group (NLS group)/LS group (LS group)

Table S7. Logistic regression analysis of the important factors in the 2022E group in the E group

E group	B	SE	Wald	df	p	Exp(B)	95% CI
Sex	0.023	0.635	0.001	1	0.971	1.023	0.295–3.554
Age	0.070	0.040	3.109	1	0.078	1.072	0.992–1.159
2018PF	0.020	0.031	0.411	1	0.521	1.020	0.959–1.085
2022PF	0.018	0.028	0.408	1	0.523	1.018	0.964–1.075
2022RP	0.005	0.026	0.039	1	0.843	1.005	0.956–1.057
2022VT	-0.016	0.028	0.328	1	0.567	0.984	0.932–1.039
2022SF	0.021	0.013	2.503	1	0.114	1.021	0.995–1.047
2022RE	0.002	0.026	0.004	1	0.948	1.002	0.952–1.054
2022MH	-0.015	0.029	0.265	1	0.607	0.985	0.931–1.043
2022EQ-5D-5L	1.108	2.974	0.139	1	0.709	3.030	0.009–1029.44
2022LS	-2.092	0.912	5.260	1	0.022	0.123	0.021–0.738

E group, the group without exercise habits in 2018; 2022E group, the group with exercise habits in 2022; PF, physical functioning; RP, physical role; VT, vitality; SF, social functioning; RE, emotional role; MH, mental health; EQ-5D-5L, EuroQoL 5-dimension 5-level; LS, locomotive syndrome.
